# 

**Published:** 1912-04-20

**Authors:** 


### APPOINTMENTS.

The following appointments at the Royal Infirmary, Sunderland, have been made: Dr. W. R. Ridley, house physician; Dr. P. Vieyra, junior house surgeon; and Dr. Margaret B. S. Daroch, resident medical officer for the Children's Hospital.

### OUR BUREAU OF INFORMATION.

### Inexpensive Homes for Mild Mental Cases.

There is hardly a more pressing need among middle-class people than that for accommodation at reasonable rates for persons ill adapted for the struggle for existence. It would be instructive to learn exactly the percentage of the population requiring segregation without being eligible for asylum treatment. The number of children in this condition who are unable to earn their own living, and whose parents are unable to make any provision for them, has been estimated recently at 20,000. Many of these defectives die before the age of twenty-one. Many are provided for in refuges and gaols into which they tend to drift, not from any really vicious tendencies, but for want of ability to live the ordinary life of society and follow any regular employment. The promised measure for dealing with defective children and adults may be expected to bring about an amazing reduction in the number of these persons in the next generation. But it is the large section of persons not quite normal yet not eligible for public assistance with which we are now concerned. Their relatives shrink from confining them in asylums, and, indeed, many persons who fall under this description could not be certified as insane. They are to be encountered very often in private families, casting a gloom over the home circle from faults of unrestrained temper, or from intemperance, or from other failings which call for watchful care and skilled management, yet do not debar them from enjoying life in their own way. We have known the entire childhood of boys and girls warped through the presence of some uncle or aunt in the family who was not entirely responsible for the ill-will provoked by their very presence. Every worker knows instances of the painful dilemma occasioned by the want of suitable homes for this class of patient. The ordinary terms for even "mild" mental patients are prohibitive. Middle-class families cannot afford to lay aside as much as £200 a year out of a professional income for the incapable relative. They are compelled to put up with the infliction as best they may.

A week or two back we were asked through the Information Bureau to name a home in which such a patient could be received on very moderate terms. The number of replies we have received convinces us that there is a wide range of nursing homes managed on economical but sound lines which are able to receive occasional patients requiring watchful care at a guinea a week to fill vacant beds. No home could possibly pay its way solely on patients contributing so small a sum. And hence they are chary of advertising their willingness to make reductions in their usual rates. But the advantage of the Information Bureau is apparent in that it exactly fills the office of placing in communication those who need the reduction and those who are prepared to make it. Will our readers make it known that we have a good selection of homes prepared to make large reductions to suitable cases where means cannot be found to pay the full terms? There can be no doubt that the need for cheap accommodation of this particular kind is rapidly increasing and ought to be met.

### NEEDS AND HELPERS.

### Hospitals in Bahia Blanca, Argentina.

"Dr. D. R., Bristol": There is a municipal hospital at Bahia Blanca, under the charge of Dr. N. S. Mallea. We have ascertained that there is no British hospital in that port.

### Home for a Mild Mental Case.

Will "A B C" communicate with Miss Martin, "Clovelly," Kedleston Road, Derby, who can offer very suitable accommodation at the terms named for this patient?

"Sunshine"; "Nurse Foster"; "Nurse Murray"; "Mrs. Scales"; "Mrs. Allan" (six stamps received): By complying with Rule 6 (see below) the homes, which all appear very suitable for the case mentioned, can be placed on our List of Approved Homes, and will be brought to the notice of correspondents requiring accommodation of this nature. Full particulars should be given of the situation, etc., of the home, the class of cases received, and the terms; mention should be made if circumstances admit from time to time of any reduction in the usual charges. Two references must be supplied. Particulars of homes when thus registered will be inserted in The Hospital, and in the quarterly list of recent additions to approved homes.

### Training as Almoner.

"Nurse Mayall": There is no regular school for training hospital almoners, but if you apply to the Hospital Almoners' Council, Denison House, Vauxhall Bridge Road, London, you will receive information as to the best way to prepare for undertaking this branch of work.

### Further Experience for a Certified Midwife.

"Nurse Mayall": Perhaps one of our readers may be able to put you in the way of getting a few weeks' additional experience in a hospital, especially as you hold the C.M.B. certificate and require no salary. Are you a fully trained nurse? If so, you might apply for a holiday appointment.

### Rules for Correspondents.

Everyone who uses or wishes to help our Bureau of Information must be kind enough to observe the following rules:—

(1) Postal replies cannot be given. Exception will only be made in very special cases at the Editor's discretion.

(2) Queries or answers relating to different cases must be written, or preferably typed, on separate sheets of paper, and the writing must never be crossed.

(3) Questions and replies received not later than Wednesday will, as far as possible, be dealt with in our issue of the following Saturday week.

(4) Letters must have attached the name and address of the sender, with a pseudonym for publication if preferred.

(5) Applications referring to non-dependents where the need relates to business or employment must be accompanied by a postal order for 5s., when the requirement shall have publicity in these columns.

(6) All applicants for insertion in our list of Approved Homes must send a registration fee of 5s. The omission of this leads to delay and gives avoidable trouble.

(7) *All communications* for this department *must be accompanied by the coupon* to be found at the bottom of the third page of the cover on the inside, and should be addressed to the Editor of Bureau of Information, c/o The Hospital, 29 Southampton Street, Strand, London, W.C.

### Florence Nightingale Memorial.

The following contributions have been received during the past week by Mr. G. Q. Roberts: A. Mortimer Singer, Esq., £100; Lady Phillips, £20; R. B. Angus, Esq., £10; Lord Milner, £10; the Dowager Countess of Strathmore, £5; Officers, Non-commissioned Officers, and Men, 1st Norfolk Regiment, £3 1s. 8d.; Corps of Military Police (additional), £2 7s.; Nurse F. James's collection, £2 3s.; Lieut.-Col. Sir Henry Trotter, K.C.M.G., £2 2s.; Lady Alice Lowther, £2 2s.; Lady Trotter, £1 1s.; Mrs. Currey (annuities), £1 1s.; Sir Courtenay P. Ilbert, K.C.B., £1 1s.; "In Memory of Mrs. Fanny Lovsey," £1 1s.; C. W. Bennett, Esq., £1; Nurses' Union (London Division), 13s.; Staff of Station Hospital, Secunderabad, 12s. 6d.; Nurses' Union (additional), 6s.

